# Supplementary material for: Cu(Proline)2 Complex: A Model of Bio-Copper Structural Ambivalence
Source: Molecules. 2022 Sep 9;27(18):5846. doi: 10.3390/molecules27185846 (PMC9502899; doi:10.3390/molecules27185846)
Supplement: Supplementary file 1 [file molecules-27-05846-s001.zip › molecules-1853542-supplementary.pdf]

Supplementary Materials

Dynamics of a Cu(Proline)$_2$ complex: a model of bio-copper structural ambivalence.

Victor V. Volkov[1], Riccardo Chelli[2], Carole C. Perry[1*]

[1]Interdisciplinary Biomedical Research Centre, School of Science and Technology, Nottingham Trent University, Clifton Lane, Nottingham NG11 8NS, United Kingdom.
[2]Dipartimento di Chimica, Università di Firenze, Via della Lastruccia 3, I-50019 Sesto Fiorentino, Italy.

Here, we present details to complement and support the experimental sections and discussion. We provide descriptions of: 1) *ab initio* molecular dynamics; 2) energies along the trajectories simulated for the considered complexes; 3) structural properties simulated for the complexes; 4) atomic coordinates for the structures of the complex (ground state and first excited state) under different degrees of hydration used for DFT studies given in pdb format; 5) results of time dependent studies of deuterated water stretching; and references.

1. *Ab initio* molecular dynamics

To model structural and electronic properties of Cu(L-proline)$_2$ complex in aqueous solution we use ab initio molecular dynamics (MD) code implemented in CP2K package [1]. Specifically, to account physics of the complex (where the copper ion has an unpaired electron) and its hydration, we simulate NVE Born–Oppenheimer (BO) MD trajectories using optimized contracted Gaussian basis sets of double-zeta valence polarized quality, augmented with diffuse primitives [2] under the density functional of Perdew, Burke and Ernzerhof [3]: The model structural cases are presented in **Figure S1**.

The simulations for the complexes are conducted starting at 300 K with time-step 0.2 fs. Target accuracy for the self-consistent field convergence is 1.00E-06 and cut-off of the fine grid level was set at 400 Rydberg. Consistently, for higher accuracy, we define the grid to map a Gaussian setting relative cut-off to 100 Rydberg. Energy correction accuracy threshold is 10^{-12}. The number of additional molecular orbitals for each spin is set to 100. Simulations of the system under various hydrations are conducted in a box 17x17x17 Angstrom under periodic boundary conditions.

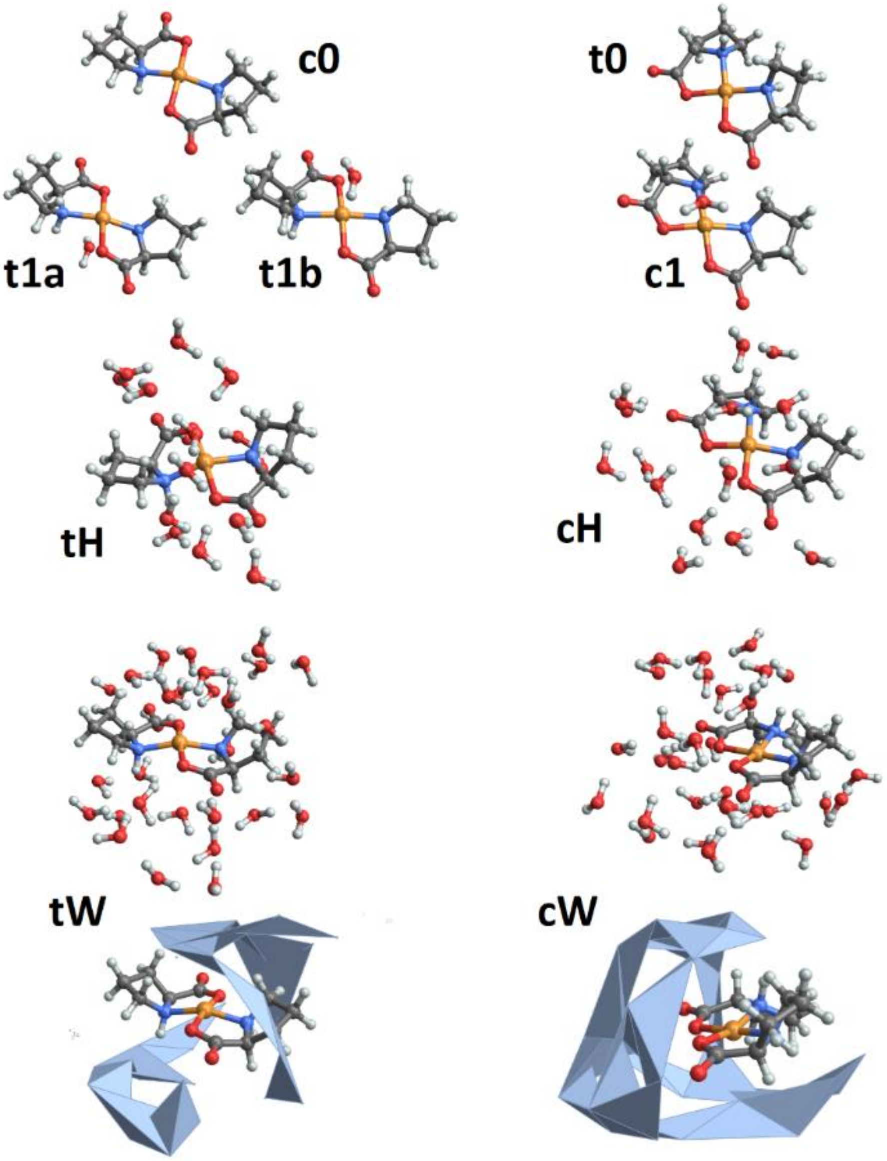

Figure S1. **Model systems explored using BOMD and DFT studies: cis (c) and trans (t) complexes:** without water, structures **c0** and **t0**; with 1 water at copper ion: structural cases **c1**, **t1a** (where the water on the side of the NH groups)and **t1b** (where the water on the side of the CH groups); with 15 water molecules: structural cases **cH** and **tH**; and with 29 water molecules: structural cases **cW** and **tW**. Here, in the figure, for the latter cases, we use a convex hull presentation for each water molecule to contrast visually the tendencies of waters to distribute around the complex under the two geometries.

2. Energies along the trajectories simulated for the considered complexes

Figures S2 and **S3** show the Kinetic and Potential energies for the system over 500 fs. The energy samplings suggest that, while it takes 10-20 fs for the dry systems to equilibrate, introduction of water, as expected increases the time to equilibration significantly.

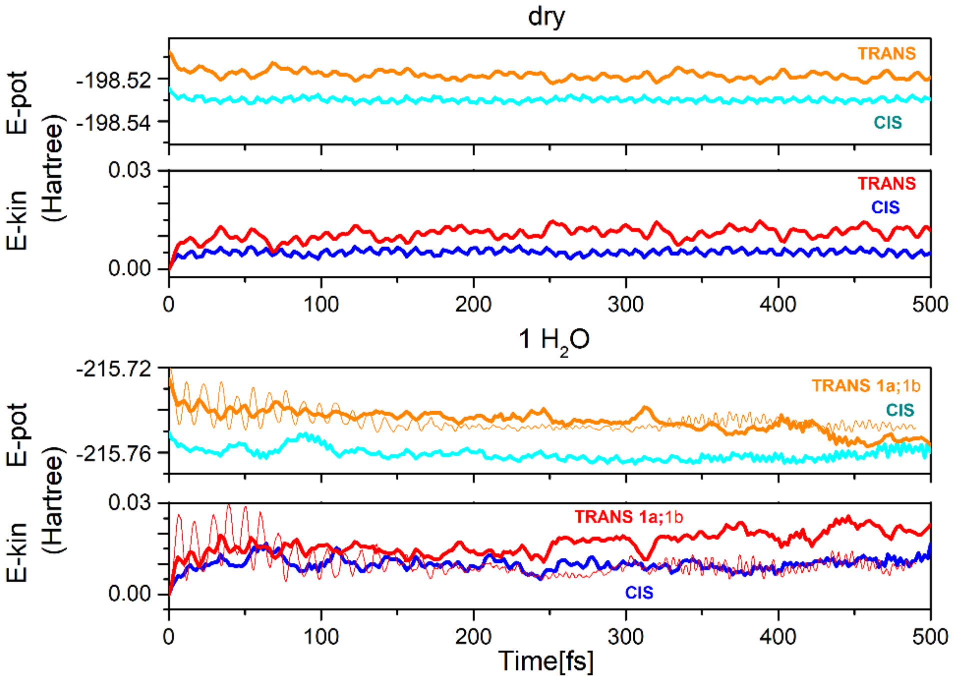

Figure S2. **Kinetic and potential energies upon initialization and development of BOMD trajectories** for **cis** and **trans** complexes without water: structures **c0** and **t0**; and with 1 water molecule associated with the copper ion: structural cases **c1**, **t1a**, and **t1b**.

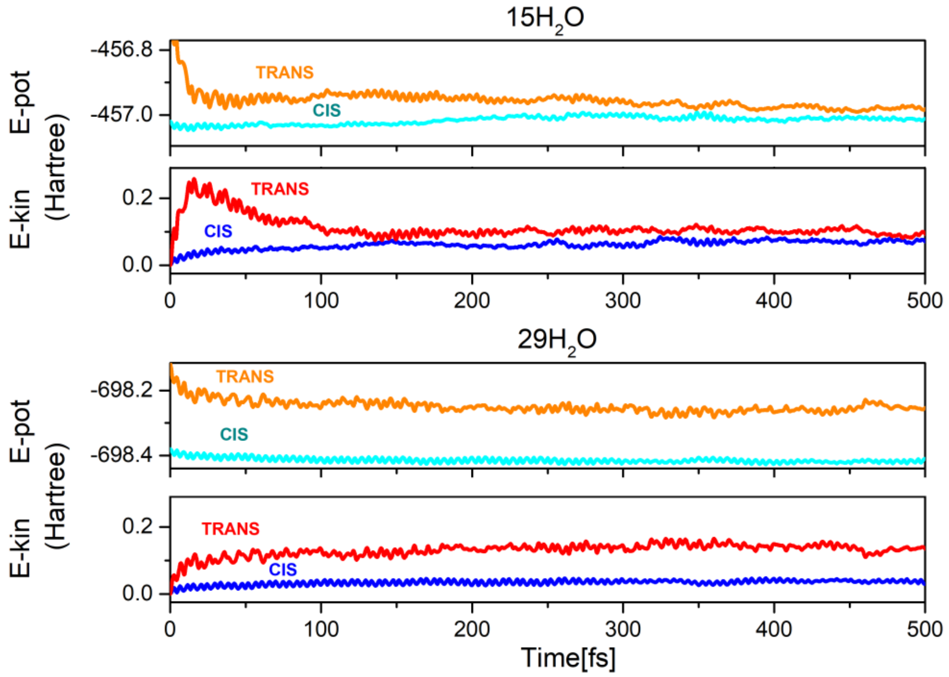

Figure S3. **Kinetic and potential energies upon initialization and development of BOMD trajectories** for **cis** and **trans** complexes with 15 waters: structural cases **cH** and **tH**; and with 29 water molecules: structures **cW** and **tW**.

Theory simulates a strong oscillatory pattern for the energies in the case **t1b**. The oscillations reflect settlement of water above the copper ion and between the hydrogens of the CH moieties of the proline side groups. Upon completion of the simulations the energies of all structures under *cis* and *trans* geometries become comparable, save for the cases of **cW** and **tW**. The energy differences there may be readily attributed to a more extended hydrogen bonded water network formed for the cis complex: see **Figure S1** which allows an extended aqueous shell around them. Under the *trans* geometry, the same number of waters are distributed more discretely: see **Figure S1**. At the same time, the complexity of the more fragmentary aqueous network in **tW** suggests an entropic gain. Here, the energetic differences for the **cW** and **tW** are due to aqueous structuring.

Accounting equilibration time window, next, we choose representative **c0**, **t0**, **t1b**, **cH** and **tH** structural cases for DFT structural optimization, computation of normal modes, anharmonicities and TD-DFT evaluation of the electronic properties of these systems. DFT calculations were performed using the Gaussian 09 program [4]. The complexes are in a doublet spin state and hence they have been treated through the unrestricted B3LYP functional. Considering earlier studies in similar systems [5-7,8], the LANL2DZ basis set is employed for the Cu ion, whereas the 6-31++g(d,p) basis set is used for all other atoms.

In the main text, we give less attention to the structural properties of the structural case **t1a** because both, BOMD simulations and DFT optimizations indicate that when the **trans** complex is in the presence of several water molecules, the copper ion follows coordination with water on the side of the CH terminal moieties rather than on the side of the NH moieties. Structural cases **cW** and **tW** were excluded for DFT studies because, at present, the size of the systems is beyond computational capacity.

None the less, as we present in our structural analysis, DFT optimizations of **tH** and **cH** structural cases, where interactions with the first neighbour waters are accounted explicitly, demonstrate reasonable agreement with averaged structural tendencies simulated for the systems using the BOMD approach when we instruct g09 to account for the bulk solvent phase using the polarizable continuum model (PCM) [9].

3. Structural properties simulated for the considered complexes

In particular, in **Figures S4** and **S5** we compare the dynamics of departure of the complex from planar geometry (see blue and red lines) with the results of structural optimizations for the corresponding systems using DFT (see magenta and orange points). The results shown in the panels (**c** and **d**) indicate a reasonable agreement of the ring departure from planar after DFT optimization for tH, and cH with average characteristics of BOMD simulated **tH**, **cH**, **tW** and **cW**. Here, it is interesting to note that data in panels (**a1-d1)** in **Figures S4** and **S5** suggest that, upon adding more water, one of the cyclic components departs from ideal planar geometry more than the other. This may correlate with the fact that water, which coordinates with copper while above it, may not participate with the aqueous network around both carbonyls identically (symmetrically). Therefore, the difference in departure from planarity for the two cyclic components of the complex may reflect the difference in hydration of the two carbonyls.

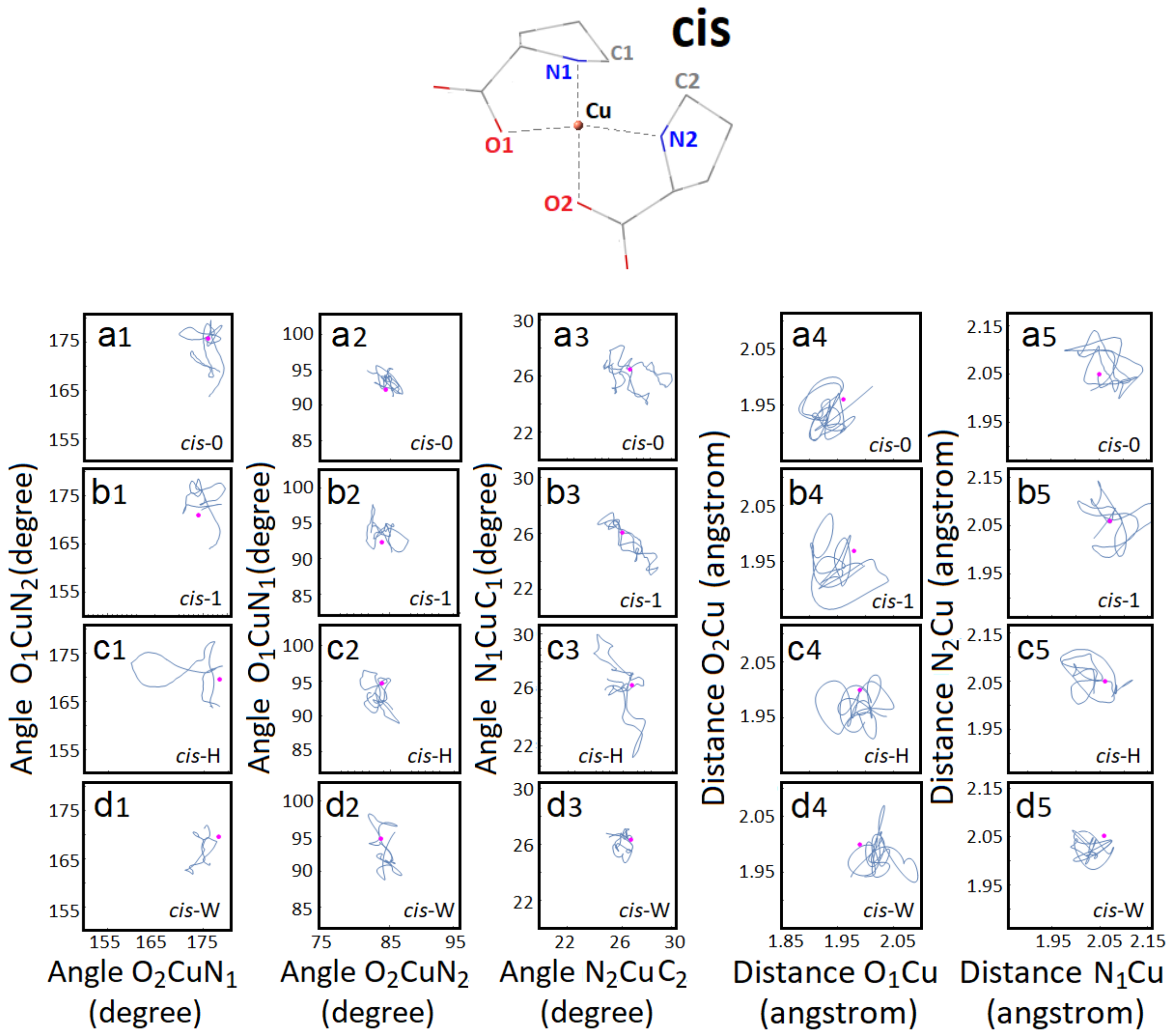

Figure S4. **Dynamics of the rings in the plane: cis complex. Top:** graphical definitions of the geometric properties in the simulated structures. For a cis complex without water (the structural case cis-0), panel (**a1**): variance of the O1-Cu-N2 angle mapped on the variance of the O2-Cu-N1 angle; panel (**a2**): variance of the O1-Cu-N1 angle mapped on the variance of the O2-Cu-N2 angle; panel (**a3**): variance of the N1-Cu-C1 angle mapped on the variance of the N2-Cu-C2 angle; panel (**a4**): variance of the O1-Cu bond length mapped on the variance of the O2-Cu bond length; panel (**a5**): variance of the N1-Cu bond length mapped on the variance of the N2-Cu bond length. Panels (**b, c** and **d)** show analogous data for a cis complex with 1 water (structure cis-1), with 15 waters (structure cis-H) and 29 water molecules (structure cis-W), respectively. Magenta dots present results of DFT structural optimization for the corresponding cis-0, cis-1, and cis-H structural cases. Magenta dots in panels (**d**) show results of DFT structural optimization for the structural case cis-H.

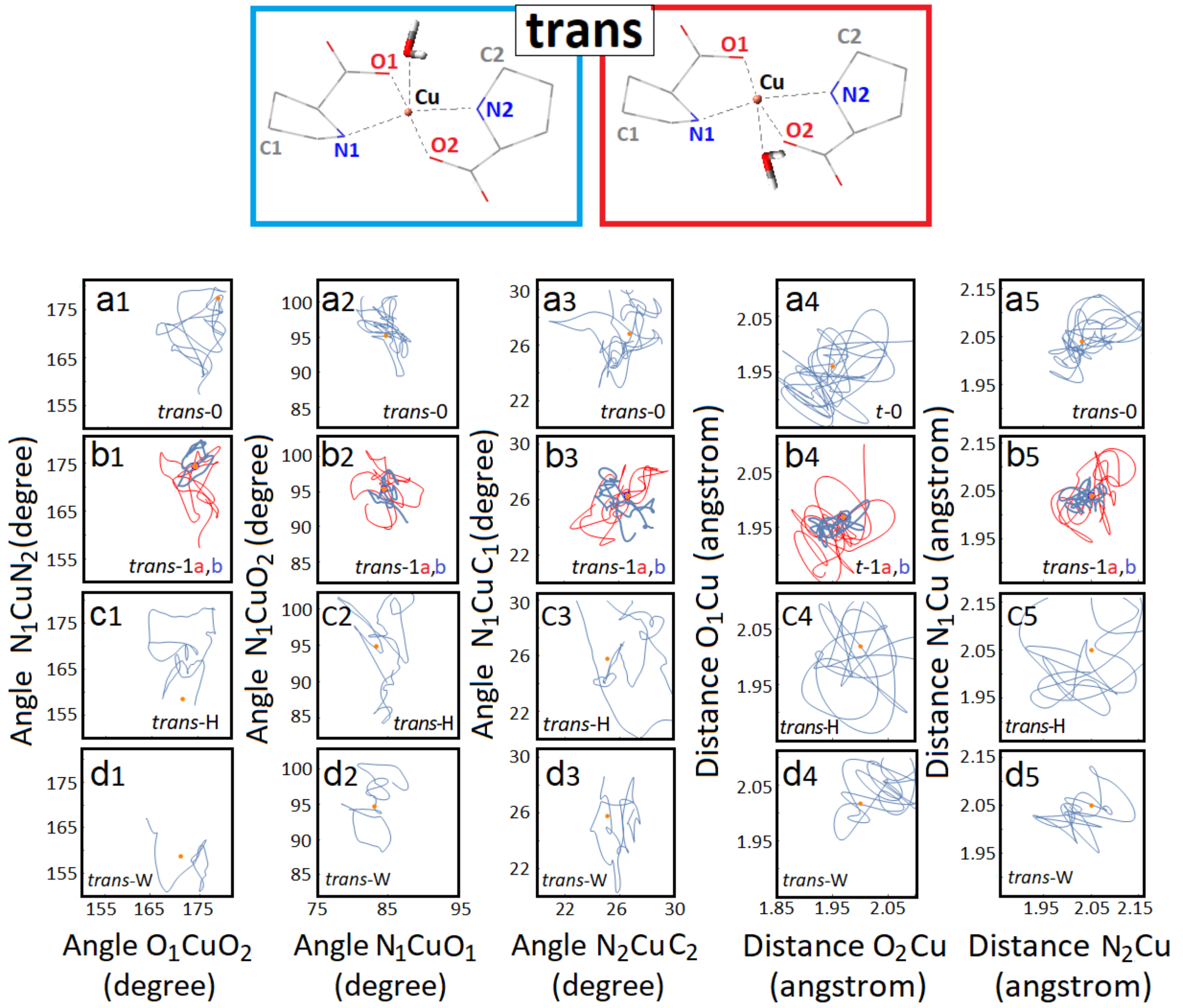

Figure S5. **Dynamics of the rings in the plane: trans complex. Top:** graphical definitions of the geometric properties in the simulated structures. For the **trans** complex without water (the structural case **t0**), panel (**a1**): variance of the N1-Cu-N2 angle mapped on the variance of the O1-Cu-O2 angle; panel (**a2**): variance of the N1-Cu-O2 angle mapped on the variance of the N1-Cu-O1 angle; panel (**a3**): variance of the N1-Cu-C1 angle mapped on the variance of the N2-Cu-C2 angle; panel (**a4**): variance of the O1-Cu bond length mapped on the variance of the O2-Cu bond length; panel (**a5**): variance of the N1-Cu bond length mapped on the variance of the N2-Cu bond length. Panels (**b**) presents analogous data using a red line for the **trans** complex with 1 water on the side of NH groups (structure **t1a**), and using a blue line for the **trans** complex with 1 water on the side of CH groups (structure **t1b**). Panels (**c** and **d**)show analogous data for the **trans** complex with 15 waters (structure **tH**) and 29 water molecules (structure **tW**), respectively. In these structures water shows a better coordination with the copper while on the side of CH group, as under the **t1b** geometry. Orange dots present results of DFT structural optimization for the corresponding **t0**, **t1**, and **tH** structural cases. Note that orange dots in D present results of DFT structural optimization for the structural case **tH**.

Next, in **Figures S6** and **S7** we present information on the dynamics of water, which coordinates copper ion apically in the initial geometry. Simulations for *cis* geometry suggest a conservative character of the water dynamics – overall, the apically coordinated water does not lose its site. When alone (structural case **c1**) it is located more distantly from copper, compared to when in hydrated structural cases, **cH** and **cW**.

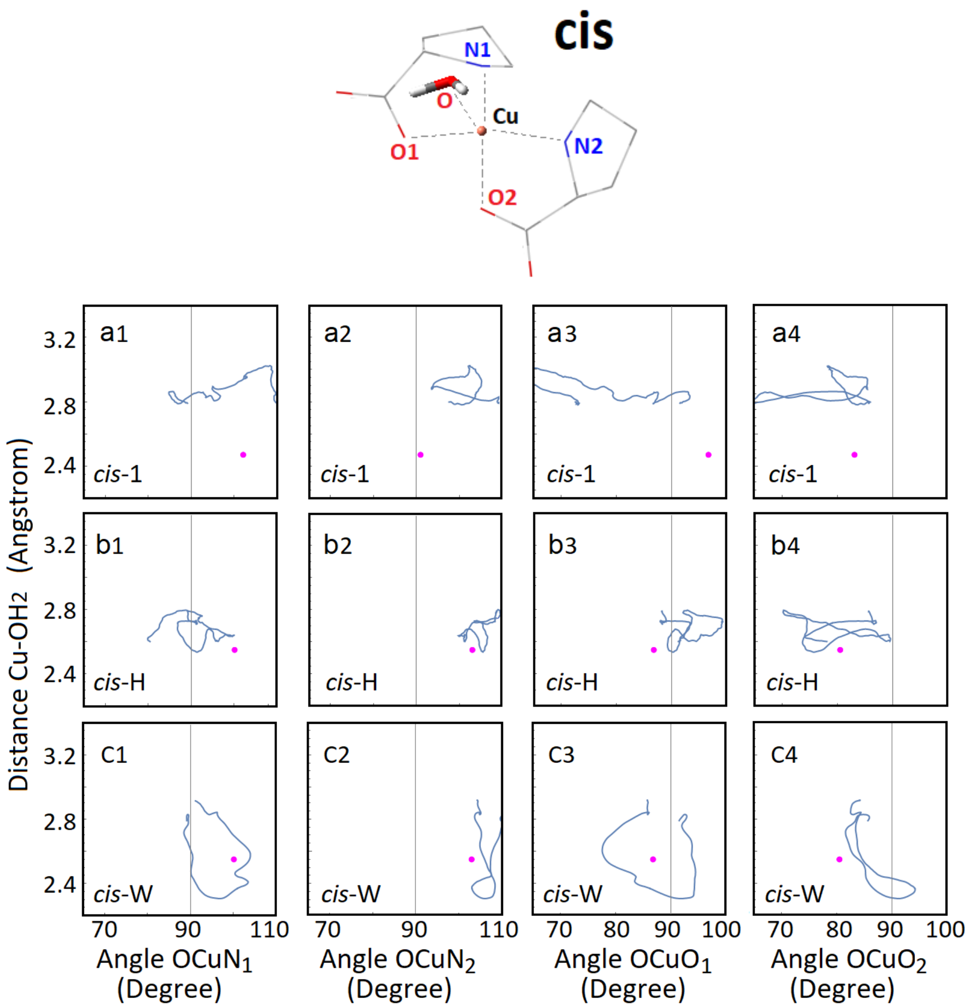

Figure S6. **Dynamics of the water-Cu^{2+} coordination: cis complex. Top:** graphical definitions of the geometric properties in the simulated structures. For a **cis** complex with one water (the structural case **c1**): panel (**a1**): variance of the O-Cu bond length mapped on the variance of the O-Cu-N1 angle; panel (**a2**): variance of the O-Cu bond length mapped on the variance of the O-Cu-N2 angle; panel (**a3**) variance of the O-Cu bond length mapped on the variance of the O-Cu-O1 angle; panel (**a4**): variance of the O-Cu bond length mapped on the variance of the O-Cu-O2 angle. Panels (**b** and **c**) present analogous data for a **cis** complex with 15 waters (structure **cH**) and 29 water molecules (structure **cW**), respectively. Magenta dots present results of DFT structural optimization for the corresponding **c1**, **c2**, and **cH** structural cases. Note that magenta dots in panels (**c**)present results of DFT structural optimization for the structural case **cH**.

Simulations of water coordination to copper for *trans* geometry systems suggest less tight coordination. In the structural case **t1a**, when the single water is on the side of the NH terminal moieties coordination is relatively stable. When in the structural case **t1b**, facing the CH terminals, water may lose its site. In the case of a larger aqueous cluster in **tH**, coordination with copper happens on the side of the CH groups. However, structural dynamics of the apically coordinated water there is less conserved: panels (**b**) in **Figure S7** show that such water may leave its place giving the position to another water. Structural stability of the apically coordinated water for both, cis and trans geometries is established in the relatively well hydrated systems **cH** and **tH**: see panels (**c**) in **Figures S6** and **S7**.

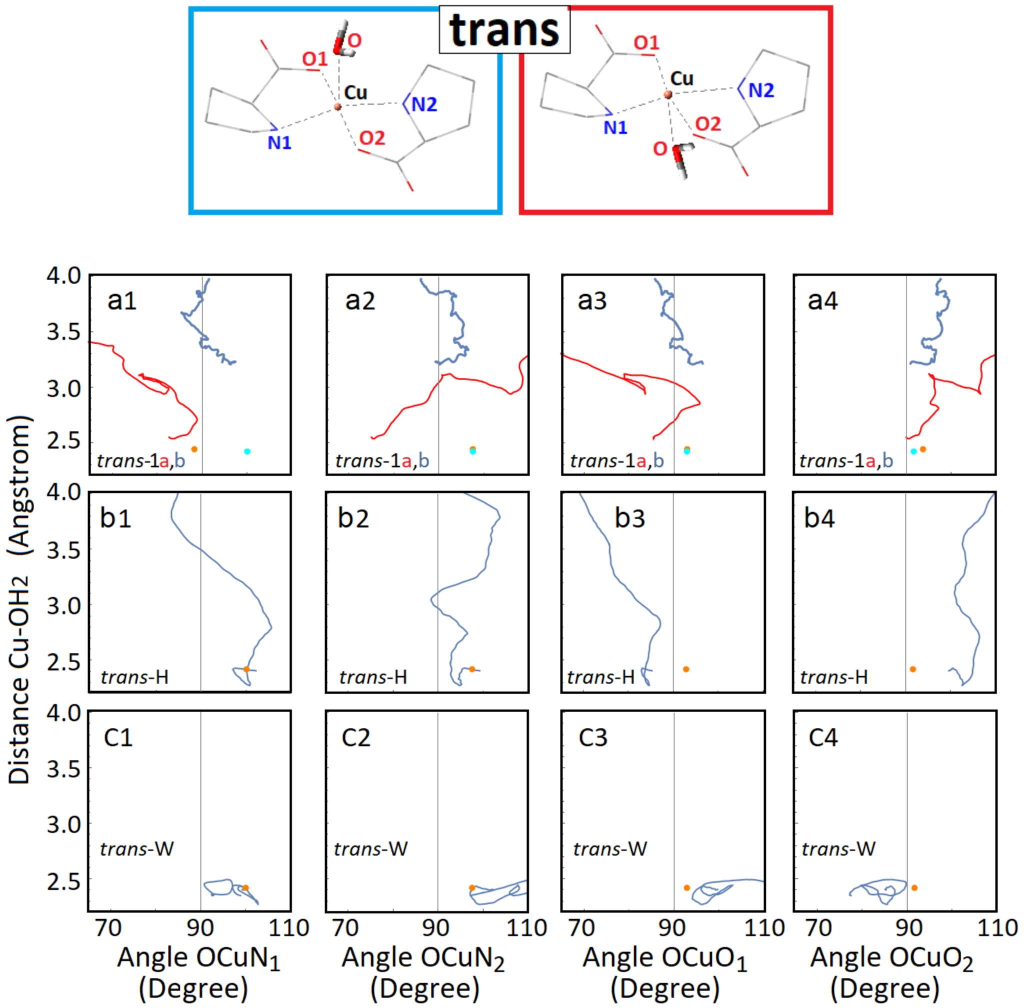

Figure S7. **Dynamics of the water-Cu^{2+} coordination: trans complex. Top:** graphical definitions of the geometric properties in the simulated structures. For the structure **t1a** (red line), and for the structure **t1b** (blue line), panel (**a1**): variance of the O-Cu bond length mapped on the variance of the O-Cu-N1 angle; panel (**a2**): variance of the O-Cu bond length mapped on the variance of the O-Cu-N2 angle; panel (**a3**): variance of the O-Cu bond length mapped on the variance of the O-Cu-O1 angle; panel (**a4**): variance of the O-Cu bond length mapped on the variance of the O-Cu-O2 angle . In panels (**a**): the set of orange and cyan dots present results of DFT structural optimization for the structures **t1a** and **t1b**, respectively. Panels (**b** and **c**) present analogous data for **trans** complex with 15 waters (structure **tH**) and 29 water molecules (structure **tW**), respectively. Here, orange dots present results of DFT structural optimization for the **tH**, and **tW** structural cases.

However, the anticipated higher mobility of water associated with a Copper complex when in a trans geometry may favour a higher entropic factor in the structural realization of this system. The results of DFT structural optimizations (as used for analysis in the main text) confirm copper hydration characteristics as BOMD for the relatively well hydrated systems **cH** and **tH**.

In **Figure S8** we present distance-angular properties to characterise hydrogen bond dynamics of water next to carbonyl moieties as simulated and **cH**, **tH**, **cH** and **cW** systems. In analogy to

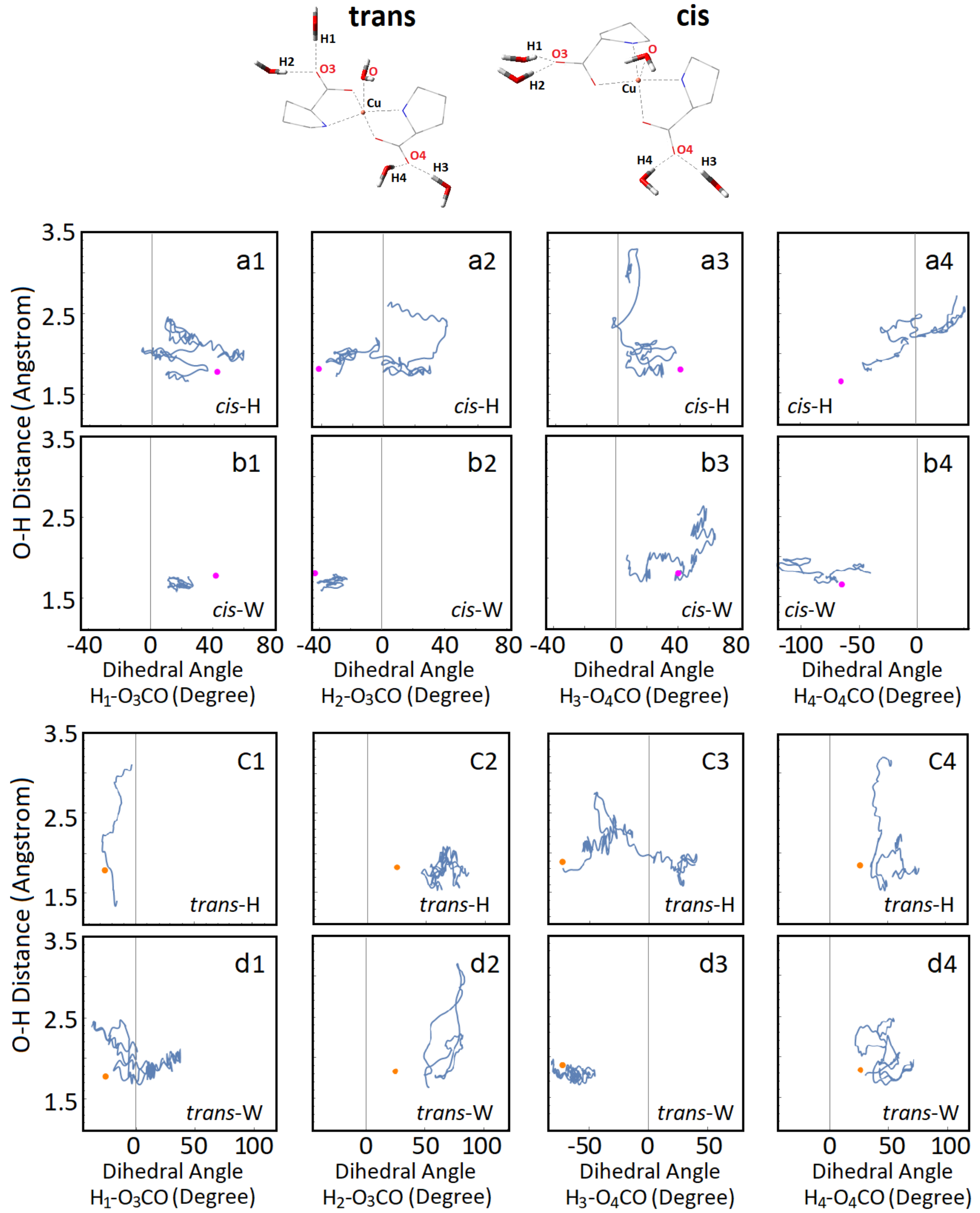

Figure S8. **Hydrogen bond dynamics at carbonyls: cis and trans complexes. Top:** graphical definitions of the geometric properties in the simulated structures. For a **cis** complex with 15 waters (the structural case **cH**): panel (**a1**): variance of the H1-O3 bond length mapped on the variance of the dihedral H1-O3-C-O angle (departure of the associated H atom from the C=C-O plane); panel (**a2**): variance of the H2-O3 bond length mapped on the variance of the dihedral H2-O3-C-O angle; panel (**a3**): variance of the H3-O4 bond length mapped on the variance of the dihedral H3-O4-C-O angle; panel (**a4**): the H4-O4 bond length mapped on the variance of the dihedral H1-O4-C-O angle. panels (**b, c** and **d**) present analogous data for the **cW**, **tH**, and **tW** structural cases, respectively. Here, magenta (orange) dots present results of DFT structural optimization for the **cH** and **cW** (**tH** and **tW**) structural cases.

the character of dynamics of water coordinated apically with copper, the data sets in panels (**a** and **c**) in **Figure S8** shows that water next to carbonyls in **cH** and **tH** are more mobile than for **cW** and **tW**: see the data sets in panels (**b** and **d**). It is important to note that BOMD simulations are able to show the variation of hydrogen bonding strength with time: for example, see the increase of the distance between the hydrogen of water and carbonyl oxygen in (**d2**) panel. Overall, exploring the trajectories reveals that in system **tW**, we may expect the number of waters coordinated to a carbonyl to vary from 1 to 3.

Using BOMD trajectories we extracted **c0**, **c1**, **cH**, **t0**, **t1(a,b)**, and **tH** representative structural cases to optimize under DFT for the theoretical spectral studies as reported in the main text. Optimization of **cW** and **tW** systems is out of the contemporary hardware and software capacity to discuss harmonic and anharmonic constants. However, as one can clearly see in **Figures S4-S8**, DFT optimised representative cases **tH** and **cH** demonstrate structural properties which are very proximal to those of **tW** and **cW**, respectively.

4. Atomic coordinates for the structures of the complex under different degrees of hydration used for DFT studies given in pdb format

PDB of cis-1 in the <u>electronic ground state</u>.

```
HEADER
HETATM   1 C    2   2    5.784  24.538  25.411
HETATM   2 N    2   2    5.168  23.947  26.656
HETATM   3 C    2   2    5.228  22.451  26.529
HETATM   4 C    2   2    6.379  22.190  25.555
HETATM   5 C    2   2    6.219  23.333  24.542
HETATM   6 CU   2   2    3.303  24.782  26.946
HETATM   7 O    2   2    3.670  25.696  25.240
HETATM   8 C    2   2    4.823  25.495  24.692
HETATM   9 O    2   2    5.199  26.027  23.636
HETATM  10 N    2   2    2.948  24.115  28.863
HETATM  11 C    2   2    3.923  24.540  29.921
HETATM  12 C    2   2    3.098  24.579  31.209
HETATM  13 C    2   2    1.760  25.162  30.728
HETATM  14 C    2   2    1.580  24.574  29.304
HETATM  15 C    2   2    0.961  25.564  28.306
HETATM  16 O    2   2   -0.119  26.100  28.595
HETATM  17 O    2   2    1.604  25.764  27.202
HETATM  18 O    2   2    1.811  23.044  26.021
HETATM  19 H    2   2    5.362  22.003  27.516
HETATM  20 H    2   2    4.281  22.096  26.114
HETATM  21 H    2   2    6.660  25.123  25.708
HETATM  22 H    2   2    5.437  23.088  23.815
HETATM  23 H    2   2    7.133  23.553  23.986
HETATM  24 H    2   2    6.314  21.200  25.094
HETATM  25 H    2   2    7.342  22.263  26.074
HETATM  26 H    2   2    2.943  23.097  28.821
HETATM  27 H    2   2    4.766  23.847  29.948
HETATM  28 H    2   2    4.298  25.538  29.673
HETATM  29 H    2   2    2.963  23.564  31.601
HETATM  30 H    2   2    3.571  25.185  31.987
HETATM  31 H    2   2    0.920  24.903  31.376
HETATM  32 H    2   2    1.817  26.254  30.678
HETATM  33 H    2   2    0.922  23.700  29.332
HETATM  34 H    2   2    5.743  24.221  27.450
HETATM  35 H    2   2    1.977  22.624  25.165
HETATM  36 H    2   2    1.063  23.641  25.869
```

PDB of cis-1 in the <u>first electronic excited state</u>.

```
HEADER
HETATM   1 C    2   2    2.249   1.672   1.173
HETATM   2 N    2   2    1.717   1.038  -0.080
HETATM   3 C    2   2    2.830   0.300  -0.753
HETATM   4 C    2   2    3.973   0.264   0.287
HETATM   5 C    2   2    3.777   1.576   1.060
HETATM   6 CU   2   2   -0.007  -0.387   0.127
```

HETATM 7 O 2 2 1.275 -1.564 -0.904
HETATM 8 C 2 2 2.429 -1.102 -1.252
HETATM 9 O 2 2 3.248 -1.715 -1.958
HETATM 10 N 2 2 -1.660 1.009 0.244
HETATM 11 C 2 2 -2.969 0.291 0.406
HETATM 12 C 2 2 -3.887 0.824 -0.726
HETATM 13 C 2 2 -3.284 2.192 -1.074
HETATM 14 C 2 2 -1.782 1.925 -0.937
HETATM 15 C 2 2 -2.836 -1.246 0.392
HETATM 16 O 2 2 -3.888 -1.904 0.508
HETATM 17 O 2 2 0.269 -0.961 2.044
HETATM 18 O 2 2 -1.659 -1.746 0.274
HETATM 19 H 2 2 1.874 2.694 1.259
HETATM 20 H 2 2 1.891 1.110 2.041
HETATM 21 H 2 2 3.161 0.860 -1.634
HETATM 22 H 2 2 3.852 -0.595 0.958
HETATM 23 H 2 2 4.949 0.180 -0.194
HETATM 24 H 2 2 4.267 1.574 2.038
HETATM 25 H 2 2 4.171 2.420 0.482
HETATM 26 H 2 2 -1.499 1.577 1.073
HETATM 27 H 2 2 -1.176 2.819 -0.776
HETATM 28 H 2 2 -1.409 1.412 -1.831
HETATM 29 H 2 2 -3.600 2.952 -0.349
HETATM 30 H 2 2 -3.557 2.539 -2.075
HETATM 31 H 2 2 -4.929 0.877 -0.404
HETATM 32 H 2 2 -3.840 0.159 -1.595
HETATM 33 H 2 2 -3.391 0.555 1.380
HETATM 34 H 2 2 1.391 1.771 -0.704
HETATM 35 H 2 2 1.117 -1.406 2.207
HETATM 36 H 2 2 -0.420 -1.647 2.116

PDB of trans-1 in the electronic ground state.

HEADER
HETATM 1 C 2 2 3.592 24.180 28.904
HETATM 2 N 2 2 2.443 24.956 28.332
HETATM 3 C 2 2 1.871 25.814 29.428
HETATM 4 C 2 2 2.709 25.497 30.694
HETATM 5 C 2 2 3.297 24.107 30.405
HETATM 6 CU 2 2 2.878 26.128 26.711
HETATM 7 O 2 2 2.336 27.618 27.883
HETATM 8 C 2 2 1.880 27.305 29.052
HETATM 9 O 2 2 1.451 28.126 29.878
HETATM 10 N 2 2 3.498 27.319 25.167
HETATM 11 C 2 2 4.507 28.377 25.497
HETATM 12 C 2 2 5.373 28.491 24.239
HETATM 13 C 2 2 5.470 27.031 23.773
HETATM 14 C 2 2 4.062 26.457 24.072
HETATM 15 C 2 2 4.082 24.971 24.460
HETATM 16 O 2 2 4.527 24.150 23.643
HETATM 17 O 2 2 3.626 24.658 25.631
HETATM 18 O 2 2 0.697 25.986 25.621
HETATM 19 H 2 2 3.993 29.296 25.784
HETATM 20 H 2 2 5.107 28.039 26.348
HETATM 21 H 2 2 3.424 26.552 23.188
HETATM 22 H 2 2 6.228 26.497 24.358
HETATM 23 H 2 2 5.729 26.926 22.718
HETATM 24 H 2 2 6.350 28.938 24.446
HETATM 25 H 2 2 4.869 29.105 23.484
HETATM 26 H 2 2 3.666 23.214 28.403
HETATM 27 H 2 2 4.517 24.735 28.721
HETATM 28 H 2 2 2.558 23.323 30.609
HETATM 29 H 2 2 4.193 23.893 30.995
HETATM 30 H 2 2 2.097 25.531 31.597
HETATM 31 H 2 2 3.514 26.231 30.807
HETATM 32 H 2 2 0.827 25.527 29.586
HETATM 33 H 2 2 1.731 24.299 28.020
HETATM 34 H 2 2 2.644 27.762 24.835
HETATM 35 H 2 2 0.456 25.124 25.256
HETATM 36 H 2 2 -0.075 26.282 26.123

PDB of trans-1 in the first electronic excited state.

HEADER
HETATM 1 C 2 2 -2.994 -0.029 0.116
HETATM 2 N 2 2 -1.909 0.998 0.232
HETATM 3 C 2 2 -1.963 1.871 -0.982
HETATM 4 C 2 2 -3.403 1.749 -1.490

```
HETATM    5  C     2     2      -3.713   0.268  -1.228
HETATM    6 CU    2     2       0.003   0.011   0.582
HETATM    7  O     2     2      -0.121   0.016   2.592
HETATM    8  C     2     2      -2.477  -1.482   0.201
HETATM    9  O     2     2      -1.214  -1.666   0.351
HETATM   10  N     2     2       1.914  -0.997   0.239
HETATM   11  C     2     2       1.943  -1.845  -0.995
HETATM   12  C     2     2       3.383  -1.744  -1.505
HETATM   13  C     2     2       3.726  -0.275  -1.218
HETATM   14  C     2     2       3.008   0.020   0.128
HETATM   15  C     2     2       2.501   1.476   0.218
HETATM   16  O     2     2       3.355   2.380   0.133
HETATM   17  O     2     2       1.240   1.668   0.375
HETATM   18  O     2     2      -3.326  -2.391   0.119
HETATM   19  H     2     2      -1.655   2.885  -0.719
HETATM   20  H     2     2      -1.261   1.487  -1.731
HETATM   21  H     2     2      -3.694   0.101   0.948
HETATM   22  H     2     2      -3.291  -0.352  -2.027
HETATM   23  H     2     2      -4.781   0.050  -1.167
HETATM   24  H     2     2      -3.502   2.027  -2.544
HETATM   25  H     2     2      -4.067   2.394  -0.902
HETATM   26  H     2     2       1.612  -2.856  -0.754
HETATM   27  H     2     2       1.248  -1.428  -1.733
HETATM   28  H     2     2       4.035  -2.414  -0.931
HETATM   29  H     2     2       3.474  -2.004  -2.564
HETATM   30  H     2     2       4.799  -0.082  -1.150
HETATM   31  H     2     2       3.323   0.367  -2.009
HETATM   32  H     2     2       3.704  -0.118   0.961
HETATM   33  H     2     2       2.098  -1.593   1.042
HETATM   34  H     2     2      -2.082   1.570   1.055
HETATM   35  H     2     2      -0.246  -0.875   2.957
HETATM   36  H     2     2       0.642   0.406   3.047
```

PDB of cis-H in the electronic ground state.

```
HEADER
HETATM    1  C     2     2      -2.310  -2.207  -0.439
HETATM    2  N     2     2      -0.867  -2.596  -0.628
HETATM    3  C     2     2      -0.635  -3.861   0.153
HETATM    4  C     2     2      -2.014  -4.518   0.235
HETATM    5  C     2     2      -2.944  -3.314   0.443
HETATM    6 CU    2     2       0.354  -1.005  -0.167
HETATM    7  O     2     2      -1.372  -0.115   0.318
HETATM    8  C     2     2      -2.442  -0.807   0.153
HETATM    9  O     2     2      -3.585  -0.387   0.443
HETATM   10  N     2     2       2.089  -1.735  -0.978
HETATM   11  C     2     2       2.050  -2.023  -2.454
HETATM   12  C     2     2       3.460  -1.693  -2.952
HETATM   13  C     2     2       3.824  -0.455  -2.123
HETATM   14  C     2     2       3.210  -0.764  -0.734
HETATM   15  C     2     2       2.737   0.483   0.000
HETATM   16  O     2     2       3.579   1.356   0.306
HETATM   17  O     2     2       1.482   0.582   0.276
HETATM   18  O     2     2       0.678  -1.401   2.339
HETATM   19  O     2     2      -5.853  -0.555  -1.112
HETATM   20  O     2     2      -5.832   1.839  -2.437
HETATM   21  O     2     2      -3.280   2.823  -2.459
HETATM   22  O     2     2      -1.969   2.673  -0.062
HETATM   23  O     2     2       0.672   3.345   0.141
HETATM   24  O     2     2      -4.297   0.992   2.766
HETATM   25  O     2     2       3.675   2.735   2.734
HETATM   26  O     2     2       2.289  -3.595   3.015
HETATM   27  O     2     2       3.163  -4.033   0.452
HETATM   28  O     2     2       4.701   3.290  -1.408
HETATM   29  O     2     2       1.556   4.624   2.521
HETATM   30  O     2     2      -3.447   3.639   2.172
HETATM   31  O     2     2       2.189   4.578  -1.928
HETATM   32  H     2     2       0.126  -4.463  -0.343
HETATM   33  H     2     2      -0.273  -3.593   1.148
HETATM   34  H     2     2      -2.793  -2.183  -1.421
HETATM   35  H     2     2      -2.940  -3.008   1.494
HETATM   36  H     2     2      -3.978  -3.507   0.154
HETATM   37  H     2     2      -2.076  -5.245   1.049
HETATM   38  H     2     2      -2.248  -5.033  -0.704
HETATM   39  H     2     2       2.333  -2.608  -0.486
HETATM   40  H     2     2       1.756  -3.062  -2.616
HETATM   41  H     2     2       1.314  -1.370  -2.933
HETATM   42  H     2     2       4.145  -2.521  -2.733
```

```
HETATM   43  H     2     2       3.484  -1.505  -4.029
HETATM   44  H     2     2       4.898  -0.275  -2.052
HETATM   45  H     2     2       3.360   0.438  -2.555
HETATM   46  H     2     2       3.954  -1.252  -0.096
HETATM   47  H     2     2      -0.728  -2.803  -1.615
HETATM   48  H     2     2      -0.170  -1.372   2.808
HETATM   49  H     2     2       1.073  -0.525   2.476
HETATM   50  H     2     2      -5.079  -0.588  -0.507
HETATM   51  H     2     2      -6.628  -0.771  -0.576
HETATM   52  H     2     2      -4.039   0.522   1.943
HETATM   53  H     2     2      -5.238   0.807   2.891
HETATM   54  H     2     2       4.416   2.580  -0.798
HETATM   55  H     2     2       5.341   3.821  -0.915
HETATM   56  H     2     2       3.622   2.262   1.874
HETATM   57  H     2     2       4.583   3.059   2.800
HETATM   58  H     2     2       1.006   2.431   0.246
HETATM   59  H     2     2      -1.839   1.717   0.108
HETATM   60  H     2     2      -0.300   3.232   0.012
HETATM   61  H     2     2       2.896  -3.968   1.399
HETATM   62  H     2     2       2.979  -4.941   0.179
HETATM   63  H     2     2       1.754  -2.781   2.903
HETATM   64  H     2     2       2.947  -3.399   3.695
HETATM   65  H     2     2       1.627   4.174  -1.236
HETATM   66  H     2     2       3.075   4.191  -1.787
HETATM   67  H     2     2       1.164   4.258   1.701
HETATM   68  H     2     2       2.326   4.043   2.685
HETATM   69  H     2     2      -2.446   2.742  -0.925
HETATM   70  H     2     2      -2.925   3.399   1.379
HETATM   71  H     2     2      -3.816   2.784   2.469
HETATM   72  H     2     2      -3.291   3.690  -2.885
HETATM   73  H     2     2      -4.216   2.504  -2.450
HETATM   74  H     2     2      -6.486   2.431  -2.043
HETATM   75  H     2     2      -5.894   0.988  -1.936
```

PDB of cis-H in the first electronic excited state.

```
HEADER
HETATM    1  C     2     2       3.667  -0.841  -0.806
HETATM    2  N     2     2       2.446  -1.713  -0.847
HETATM    3  C     2     2       2.206  -2.118  -2.266
HETATM    4  C     2     2       3.428  -1.595  -3.061
HETATM    5  C     2     2       3.869  -0.369  -2.250
HETATM    6 CU     2     2       2.422  -3.519   0.435
HETATM    7  O     2     2       4.415  -3.899   0.572
HETATM    8  C     2     2       1.985  -3.623  -2.436
HETATM    9  O     2     2       2.044  -4.385  -1.407
HETATM   10  N     2     2       1.830  -2.660   2.312
HETATM   11  C     2     2       1.710  -3.688   3.389
HETATM   12  C     2     2       0.254  -3.590   3.918
HETATM   13  C     2     2      -0.147  -2.147   3.583
HETATM   14  C     2     2       0.520  -1.932   2.220
HETATM   15  C     2     2       2.065  -5.103   2.921
HETATM   16  O     2     2       2.085  -6.008   3.793
HETATM   17  O     2     2       2.318  -5.298   1.682
HETATM   18  O     2     2       1.752  -4.043  -3.596
HETATM   19  O     2     2      -0.289  -3.267  -5.258
HETATM   20  O     2     2      -1.998  -5.393  -5.072
HETATM   21  O     2     2      -1.704  -6.518  -2.594
HETATM   22  O     2     2       0.887  -6.942  -1.800
HETATM   23  O     2     2       1.340  -7.798   0.763
HETATM   24  O     2     2       3.196  -5.966  -5.016
HETATM   25  O     2     2       3.942  -8.065   3.773
HETATM   26  O     2     2       6.107  -1.976   2.143
HETATM   27  O     2     2       3.896  -0.813   3.336
HETATM   28  O     2     2      -0.124  -7.347   4.862
HETATM   29  O     2     2       3.347  -9.664   1.498
HETATM   30  O     2     2       2.447  -8.372  -3.695
HETATM   31  O     2     2      -0.880  -8.553   2.374
HETATM   32  H     2     2       3.518  -0.036  -0.084
HETATM   33  H     2     2       4.524  -1.435  -0.476
HETATM   34  H     2     2       1.296  -1.630  -2.632
HETATM   35  H     2     2       4.224  -2.348  -3.075
HETATM   36  H     2     2       3.163  -1.366  -4.095
HETATM   37  H     2     2       4.904  -0.076  -2.450
HETATM   38  H     2     2       3.221   0.488  -2.467
HETATM   39  H     2     2       2.552  -1.985   2.595
HETATM   40  H     2     2       0.702  -0.884   1.970
HETATM   41  H     2     2      -0.096  -2.374   1.428
```

```
HETATM   42  H     2     2       0.268  -1.450   4.320
HETATM   43  H     2     2      -1.230  -2.003   3.545
HETATM   44  H     2     2       0.198  -3.824   4.982
HETATM   45  H     2     2      -0.394  -4.294   3.384
HETATM   46  H     2     2       2.413  -3.440   4.192
HETATM   47  H     2     2       1.649  -1.165  -0.530
HETATM   48  H     2     2       4.823  -4.069  -0.295
HETATM   49  H     2     2       4.410  -4.757   1.040
HETATM   50  H     2     2       0.507  -3.459  -4.713
HETATM   51  H     2     2       0.033  -3.009  -6.132
HETATM   52  H     2     2       2.672  -5.304  -4.514
HETATM   53  H     2     2       2.944  -5.854  -5.942
HETATM   54  H     2     2       0.666  -6.838   4.582
HETATM   55  H     2     2       0.183  -7.956   5.547
HETATM   56  H     2     2       3.264  -7.350   3.757
HETATM   57  H     2     2       3.879  -8.470   4.648
HETATM   58  H     2     2       1.720  -6.969   1.124
HETATM   59  H     2     2       1.292  -6.050  -1.736
HETATM   60  H     2     2       1.117  -7.580  -0.174
HETATM   61  H     2     2       4.744  -1.136   2.957
HETATM   62  H     2     2       3.832   0.120   3.093
HETATM   63  H     2     2       5.702  -2.709   1.650
HETATM   64  H     2     2       6.780  -2.374   2.711
HETATM   65  H     2     2      -0.130  -8.299   1.798
HETATM   66  H     2     2      -0.661  -8.182   3.252
HETATM   67  H     2     2       2.635  -9.098   1.133
HETATM   68  H     2     2       3.613  -9.197   2.315
HETATM   69  H     2     2      -0.047  -6.805  -2.092
HETATM   70  H     2     2       1.879  -7.953  -3.016
HETATM   71  H     2     2       2.753  -7.617  -4.236
HETATM   72  H     2     2      -2.303  -7.272  -2.527
HETATM   73  H     2     2      -1.820  -6.149  -3.505
HETATM   74  H     2     2      -1.842  -5.996  -5.812
HETATM   75  H     2     2      -1.376  -4.635  -5.200
```

PDB of trans-H in the electronic ground state.

```
HEADER
HETATM    1  C     2     2       1.356   2.662   0.844
HETATM    2  N     2     2       1.285   1.786  -0.365
HETATM    3  C     2     2       2.690   1.387  -0.702
HETATM    4  C     2     2       3.624   2.274   0.172
HETATM    5  C     2     2       2.699   3.383   0.702
HETATM    6 CU     2     2       0.104   0.105  -0.322
HETATM    7  O     2     2       1.869  -0.838  -0.248
HETATM    8  C     2     2       2.906  -0.113  -0.487
HETATM    9  O     2     2       4.067  -0.572  -0.562
HETATM   10  N     2     2      -0.917  -1.503  -1.085
HETATM   11  C     2     2      -2.360  -1.147  -1.290
HETATM   12  C     2     2      -3.175  -2.216  -0.528
HETATM   13  C     2     2      -2.240  -3.434  -0.535
HETATM   14  C     2     2      -0.862  -2.797  -0.327
HETATM   15  C     2     2      -2.653   0.296  -0.883
HETATM   16  O     2     2      -3.834   0.687  -0.786
HETATM   17  O     2     2      -1.636   1.072  -0.680
HETATM   18  O     2     2      -0.488  -0.144   1.921
HETATM   19  O     2     2       4.997  -2.794   0.801
HETATM   20  O     2     2      -4.632   1.030   1.935
HETATM   21  O     2     2      -1.998   3.549   0.609
HETATM   22  O     2     2       6.385   0.625  -1.618
HETATM   23  O     2     2      -6.195  -0.188  -2.050
HETATM   24  O     2     2       2.375  -2.793   1.753
HETATM   25  O     2     2      -2.205  -2.158   3.019
HETATM   26  O     2     2       0.260  -1.947  -3.780
HETATM   27  H     2     2       0.483   3.315   0.870
HETATM   28  H     2     2       1.348   2.031   1.737
HETATM   29  H     2     2       2.858   1.577  -1.767
HETATM   30  H     2     2       4.029   1.687   1.002
HETATM   31  H     2     2       4.468   2.655  -0.406
HETATM   32  H     2     2       3.049   3.805   1.649
HETATM   33  H     2     2       2.614   4.200  -0.024
HETATM   34  H     2     2      -0.029  -3.397  -0.699
HETATM   35  H     2     2      -0.693  -2.584   0.731
HETATM   36  H     2     2      -2.282  -3.946  -1.504
HETATM   37  H     2     2      -2.483  -4.159   0.246
HETATM   38  H     2     2      -4.137  -2.403  -1.009
HETATM   39  H     2     2      -3.365  -1.890   0.500
HETATM   40  H     2     2      -2.585  -1.208  -2.362
```

```
HETATM   41  H     2     2     -0.485  -1.647  -2.005
HETATM   42  H     2     2      0.903   2.333  -1.144
HETATM   43  H     2     2      0.272  -0.302   2.532
HETATM   44  H     2     2     -1.006   0.626   2.275
HETATM   45  H     2     2     -5.366   0.046  -1.585
HETATM   46  H     2     2     -4.463   0.994   0.972
HETATM   47  H     2     2      5.564   0.235  -1.250
HETATM   48  H     2     2      4.717  -2.054   0.213
HETATM   49  H     2     2      5.262  -3.517   0.218
HETATM   50  H     2     2      6.759  -0.059  -2.188
HETATM   51  H     2     2     -6.671  -0.774  -1.448
HETATM   52  H     2     2     -5.351   1.665   2.057
HETATM   53  H     2     2      2.110  -2.254   0.979
HETATM   54  H     2     2      3.328  -2.959   1.590
HETATM   55  H     2     2     -2.000   2.736   0.058
HETATM   56  H     2     2     -2.826   4.007   0.416
HETATM   57  H     2     2     -1.626  -1.474   2.622
HETATM   58  H     2     2     -2.235  -1.949   3.962
HETATM   59  H     2     2     -0.088  -2.696  -4.283
HETATM   60  H     2     2      0.252  -1.200  -4.395
HETATM   61  O     2     2     -0.323   2.770  -2.681
HETATM   62  H     2     2     -0.636   3.672  -2.831
HETATM   63  H     2     2     -1.049   2.309  -2.227
HETATM   64  O     2     2     -2.099   1.864   2.848
HETATM   65  H     2     2     -2.007   2.600   2.206
HETATM   66  H     2     2     -3.007   1.532   2.673
HETATM   67  O     2     2      1.645  -0.853   3.512
HETATM   68  H     2     2      1.388  -1.199   4.377
HETATM   69  H     2     2      1.988  -1.624   2.992
```

PDB of trans-H in the first electronic excited state.

```
HEADER
HETATM    1  C     2     2     -1.445  -2.767   0.651
HETATM    2  N     2     2     -1.364  -1.728  -0.420
HETATM    3  C     2     2     -2.759  -1.265  -0.689
HETATM    4  C     2     2     -3.705  -2.242   0.071
HETATM    5  C     2     2     -2.804  -3.436   0.429
HETATM    6 CU     2     2     -0.060  -0.059  -0.079
HETATM    7  O     2     2     -1.979   0.897   0.084
HETATM    8  C     2     2     -2.984   0.203  -0.287
HETATM    9  O     2     2     -4.161   0.644  -0.358
HETATM   10  N     2     2      1.014   1.451  -1.143
HETATM   11  C     2     2      2.457   1.094  -1.325
HETATM   12  C     2     2      3.259   2.236  -0.665
HETATM   13  C     2     2      2.330   3.447  -0.822
HETATM   14  C     2     2      0.946   2.837  -0.570
HETATM   15  C     2     2      2.794  -0.312  -0.811
HETATM   16  O     2     2      4.000  -0.619  -0.646
HETATM   17  O     2     2      1.816  -1.117  -0.604
HETATM   18  O     2     2      0.453   0.136   1.802
HETATM   19  O     2     2     -5.140   2.611   1.297
HETATM   20  O     2     2      4.553  -0.982   2.089
HETATM   21  O     2     2      2.024  -3.545   0.694
HETATM   22  O     2     2     -6.349  -0.412  -1.730
HETATM   23  O     2     2      6.377   0.282  -1.809
HETATM   24  O     2     2     -2.465   2.766   2.076
HETATM   25  O     2     2      2.178   2.278   2.904
HETATM   26  O     2     2     -0.294   1.460  -3.835
HETATM   27  H     2     2     -0.584  -3.433   0.577
HETATM   28  H     2     2     -1.416  -2.274   1.629
HETATM   29  H     2     2     -2.940  -1.315  -1.768
HETATM   30  H     2     2     -4.085  -1.766   0.980
HETATM   31  H     2     2     -4.567  -2.519  -0.540
HETATM   32  H     2     2     -3.156  -3.979   1.311
HETATM   33  H     2     2     -2.743  -4.143  -0.406
HETATM   34  H     2     2      0.124   3.383  -1.037
HETATM   35  H     2     2      0.747   2.775   0.504
HETATM   36  H     2     2      2.392   3.849  -1.840
HETATM   37  H     2     2      2.561   4.255  -0.122
HETATM   38  H     2     2      4.233   2.371  -1.140
HETATM   39  H     2     2      3.427   2.018   0.395
HETATM   40  H     2     2      2.678   1.069  -2.401
HETATM   41  H     2     2      0.566   1.461  -2.066
HETATM   42  H     2     2     -0.991  -2.174  -1.264
HETATM   43  H     2     2     -0.312   0.301   2.440
HETATM   44  H     2     2      1.020  -0.632   2.168
HETATM   45  H     2     2      5.527   0.041  -1.382
```

HETATM	46	H	2	2	4.466	-0.941	1.112
HETATM	47	H	2	2	-5.562	-0.070	-1.251
HETATM	48	H	2	2	-4.837	1.955	0.625
HETATM	49	H	2	2	-5.514	3.352	0.803
HETATM	50	H	2	2	-6.651	0.321	-2.281
HETATM	51	H	2	2	6.808	0.893	-1.198
HETATM	52	H	2	2	5.238	-1.639	2.273
HETATM	53	H	2	2	-2.242	2.266	1.259
HETATM	54	H	2	2	-3.438	2.874	2.004
HETATM	55	H	2	2	2.090	-2.744	0.123
HETATM	56	H	2	2	2.859	-4.022	0.594
HETATM	57	H	2	2	1.617	1.591	2.501
HETATM	58	H	2	2	2.084	2.153	3.857
HETATM	59	H	2	2	-0.002	2.122	-4.477
HETATM	60	H	2	2	-0.282	0.619	-4.312
HETATM	61	O	2	2	0.435	-2.550	-2.691
HETATM	62	H	2	2	0.748	-3.448	-2.865
HETATM	63	H	2	2	1.104	-2.141	-2.112
HETATM	64	O	2	2	1.968	-1.725	2.780
HETATM	65	H	2	2	1.919	-2.513	2.191
HETATM	66	H	2	2	2.901	-1.422	2.670
HETATM	67	O	2	2	-1.477	0.715	3.497
HETATM	68	H	2	2	-1.139	0.958	4.370
HETATM	69	H	2	2	-1.915	1.527	3.120

5. Time dependent studies of deuterated water stretching

To sample time-dependent spectral response of deuterated water stretching modes in Cu(L-Proline)$_2$ solution induced upon excitation of the copper d-d transition with pulsed radiation at 600 nm, we used the same experimental approach as described in the main text.

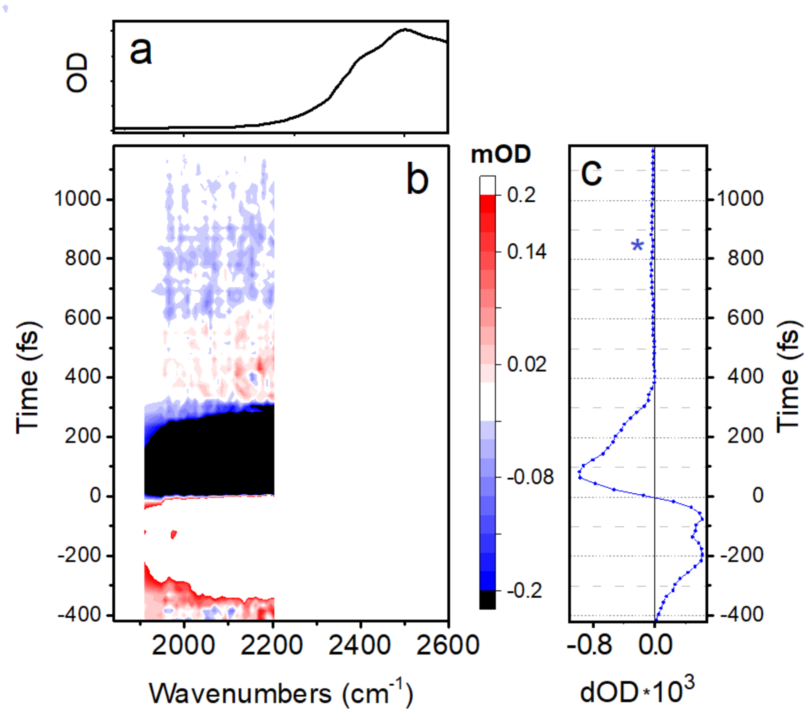

Figure S9. (**a**) FTIR spectrum of D_2O stretching modes in a thin (without spacer) sample. (**b**) Time-dependent spectral response of deuterated water stretching modes induced in a thick (70 microns spacer) sample of Cu(L-Proline)$_2$ solution using pulsed radiation at 600 nm. (**c**) Kinetic trace of D_2O stretching modes signal (from the set shown in panel **b**) at about 2100 cm^{-1}.

Specifically, after observing/measuring the signal of carbonyl modes at 1610 cm^{-1}, we adjusted the probe radiation toward the higher spectral limit of the optical amplifier (at about 2200 cm^{-1}) [10] of the spectral output of the home-made optical parametric amplifier constructed and characterized previously [11]. Following this, we sample dynamics of stretching mode involved in the deuterium bonded aqueous network, which is expected to dominate at the red side of the D_2O stretching band [12], as shown in **Figure S9a**. As we may clearly see, in **Figure S9a** and **S9c**, at zero-time, the signal is complicated with a coherent artifact. However, after 400 fs the signal shows a bleach contribution, gains its maximum value at 800 fs and decays before 1.4 ps (see the star mark in panel **c**), which we ascribe to weakening of the deuterium bond of the aqueous network next to the $Cu^{2+}(d^9)$ ion.

References

1. Kühne, T.D.; et al. CP2K: An electronic structure and molecular dynamics software package - Quickstep: Efficient and accurate electronic structure calculations. *J. Chem. Phys*. **2020**, *152*, 194103. DOI: 10.1063/5.0007045.
2. VandeVondele, J.; Hutter, J. Gaussian basis sets for accurate calculations on molecular systems in gas and condensed phases. *J. Chem. Phys*. **2007**, *127*, 114105. DOI: 10.1063/1.2770708.
3. Perdew, J.P.; Burke, K.; Ernzerhof, M.P. Generalized Gradient Approximation Made Simple. *Phys. Rev. Lett.* **1996**, *77*, 3865. DOI: 10.1103/PhysRevLett.77.3865.
4. Frisch, M.; Trucks, G.; Schlegel, H.; Scuseria, G.; Robb, M.; Cheeseman, J. et al. *Gaussian 09*, revision A.1; Gaussian, Inc.: Wallingford CT, 2009.
5. Becke, A.D. Density-functional exchange-energy approximation with correct asymptotic behavior. *Phys. Rev. A* **1988,** *38*, 3098-3100. DOI: 10.1103/PhysRevA.38.3098.
6. Dunning, T. H.; Hay, P. J. *Modern Theoretical Chemistry;* Plenum, New York, 1977, 3, pp 1-28.
7. Wedig, U.; Dolg, M.; Stoll, H.; Preuss, H. *Quantum Chemistry: The Challenge of Transition Metals and Coordination Chemistry.* Springer, 1986, pp 79-89.
8. He, Y.; Cao, X.; Nafie, L. A.; Freedman, T.B. Ab Initio VCD Calculation of a Transition-Metal Containing Molecule and a New Intensity Enhancement Mechanism for VCD. *J. Am. Chem. Soc.* **2001,** *123*, 11320-11321. DOI: 10.1021/ja016218i.
9. Tomasi, J.; Mennucci, B.; Cammi, R. Quantum mechanical continuum solvation models. *Chem. Rev.* **2005,** *105*, 2999-3093. DOI: 10.1021/cr9904009.
10. Hamm, P.; Kaindl. R.A.; Stenger J. Noise suppression in femtosecond mid-infrared light sources. *Opt. Lett.*, **2000**, *25*, 1798-1800. DOI: 10.1364/OL.25.001798.
11. Volkov, V.; Hamm, P. A Two-Dimensional Infrared Study of Localization, Structure, and Dynamics of a Dipeptide in Membrane Environment. *Biophys. J.* **2004,** *87*, 4213–4225. DOI: 10.1529/biophysj.104.045435.
12. Thämer, M.; De Marco, L.; Ramasesha, K.; Mandal A.; Tokmakoff, A. *Science* **2015**, *350*, 78-82.
